# Going green from within: correlational insights into the spread of pro-environmental behavior through the lens of organismic integration theory

**DOI:** 10.3389/fpsyg.2025.1692227

**Published:** 2025-12-12

**Authors:** Magdalena Molkenthin, Lara Christoforakos, Marc Hassenzahl, Matthias Laschke

**Affiliations:** 1 Interaction Design for Sustainability and Transformation, University of Siegen, Siegen, Germany; 2 Department of Psychology, Ludwig-Maximilians-Universität München, Munich, Germany; 3 Ubiquitous Design/Experience & Interaction, University of Siegen, Siegen, Germany

**Keywords:** pro-environmental behavior, pro-environmental motivation, organismic integration theory, extrinsic motivation, internal regulation, internalization, cross-sectoral behavior, environmental psychology

## Abstract

The share of private household's direct fuel use in global CO_2_ emissions accounts approximately 20 percent and, when indirect effects of their overall consumption are included, between 50 and 80 percent of the total resource use produced or imported by country. This underscores the importance of achieving savings through pro-environmental behavior (PEB) in all sectors of daily life, such as recycling and nutrition. As PEB is often perceived as unattractive and unexperiental, political measures using external incentives are predominantly consulted. However, these types of motivation are often short-term and context-dependent. In contrast, more internal regulated motivation not only strengthens PEB frequency but may also extend it across contexts. This occurs when the behavior aligns with personal values, is seen as personally and future-relevant, and is experienced as part of one's identity – thus helping to overcome psychological barriers in the context of environmentalism. Based on the Organismic Integration Theory (OIT), online surveys were conducted with participants from the Global North (*N* = 146), providing self-disclosure on their attitude toward environmentalism, their level of exposure to the negative consequences of climate change, their PEB in everyday life across various sectors and their type of regulation. The analysis confirmed all hypotheses, showing that higher internalized motivation was associated not only with more frequent PEB but also with its broader spread across different sectors of everyday life. Additionally, our findings indicate that the type of regulation according to OIT might be more closely associated with PEB than individuals' direct exposure to climate change, even though intrinsic motivation, as the highest level of regulation, may not necessarily need to be the focus in the context of environmentalism.

## 1 Introduction

In 2015, former US president Barack Obama stated: “We are the first generation to feel the impact of climate change, and the last generation that can do something about it. […] Our generation may not even live to see the full realization of what we do here. But the knowledge that the next generation will be better off for what we do here […] can we imagine a more worthy reward than that?” (Whitehouse, 2015).

Today, anthropogenic climate change is a tangible global challenge causing extreme weather, biodiversity loss, and health impacts (Calvin et al., 2023) that many people in the Global North still perceive as distant (Bailey, 2023; Stokes et al., 2015). Despite agreements like the Paris Climate Agreement, failing climate targets risk irreversible damages to ecosystems and society (Calvin et al., 2023), requiring immediate pro-environmental behavior (PEB) shifts. In the further course of this paper, PEB should be understood “from the actor's standpoint and covers all behaviors undertaken by a single individual to reduce one's negative environmental impact with a clear intention to change the environment [positively]” (Blankenberg and Alhusen, 2019).

Besides industry, individual consumption and household behavior play a central role in greenhouse gas emissions (Maier, 2024), with households responsible for about 20% of global CO_2_ emissions (Ivanova et al., 2016). In Europe, household emissions totaled 690 million tons in 2023 (Eurostat, 2024), with 35% direct emissions (heating, transport) and 65% indirect emissions from consumer goods (Barrutiabengoa et al., 2022). While companies are encouraged to incorporate behavioral and cultural change within organizations, they mainly rely on strategic and technological innovations to reduce their emissions. Households, on the other hand, increasingly adopt technological solutions such as energy-efficient appliances and heat pumps, however, even these strategies are accompanied by lifestyle adjustments, as the primary focus of individual mitigation remains behavioral changes, that are shaped by personal motivations and habits, such as water consumption or heating activity.

Such a behavioral change can be very challenging due to potential psychological barriers. Many individuals and households follow certain patterns in such behaviors (i.e., a certain amount of warm water consumption while showering, a certain level of heating temperature) because they are convenient, comfortable, and provide instant experiential benefits. Therefore, changing these behaviors can be unattractive and associated with sacrifice, restriction, or loss of comfort (Baxter and Pelletier, 2020; De Groot and Steg, 2010; Dioba et al., 2024; Gifford, 2011; Kollmuss and Agyeman, 2002; Nordlund and Garvill, 2002; Steg et al., 2014; Stern and Dietz, 1994). At the same time, the positive outcomes of PEB primarily benefit future generations, while the negative effects of climate change remain relatively intangible, particularly in the Global North, where households have historically experienced fewer immediate climate-related impacts compared with the Global South (Lees, 2021). Nevertheless, especially the countries of the Global North are responsible for the highest per-capita CO_2_ emissions worldwide (Ritchie et al., 2023), meaning that behavioral changes in this context have a disproportionately large potential to mitigate emissions. This stark contrast between the immediate gratification derived from energy-intensive behavior and the distant benefits of PEB creates a fundamental motivational dilemma, representing a central challenge in effectively promoting PEB within private households (Carmi and Kimhi, 2015; Spence et al., 2012). So how can individuals be motivated to engage in PEB and change their energy-intensive routines in their everyday life in the long run?

Political measures are one example to motivate PEB, using both rewards and punishments (i.e., external factors). For instance, such external factors include financial incentives such as subsidies for solar panels and electric vehicles, reduced public transport fares, as well as punishment measures such as higher taxes on petrol, diesel, and heating oil (Klimaschutzplan 2050, 2016; Bundesministerium für Wirtschaft und Energie, 2025). Although these factors can effectively initiate behavioral change (Bardach and Murayama, 2025), originating from outside the individual (i.e., they are highly extrinsically motivated) have disadvantages. For example, they only have short-term, context-dependent effects and may not work once the external factors are removed, causing relapse returning to environmentally harmful behavior (Legault, 2023; Stroebe, 2023). Heavy reliance on such external incentives may hinder the development of self-regulated motivation, as behavior remains dependent on situational reinforcement, e.g., changing government orientations, rather than internal values. Thus, motivating PEB independently from external factors remains a key challenge.

An alternative to external factors is to address internal factors that are more self-determined or fully internalized (i.e., factors originating from inside the individual) (Legault, 2023; Deci and Ryan, 1985; Pelletier et al., 2011). This does not necessarily require the achievement of intrinsic motivation, which refers to performing PEB out of pure enjoyment for the behavior itself. Instead, lasting PEB can emerge when externally regulated motivation becomes more internalized – that is, when individuals increasingly identify with the goals and values underlying PEB. However, such a change in extrinsic motivation is not an *either/or* situation, but rather a journey, in which the influence of external incentives gradually decreases, while internal reasons and personal relevance gain importance, leading to more stable and context-independent engagement (Deci and Ryan, 1985; Lepper et al., 1973; Ryan and Deci, 2000). For example, someone may begin using public transportation because of a financial incentive or tax benefit. In this case, low motivation for environmental reasons may initially be predictive as merely external factors drive the PEB (Legault et al., 2022; Schuitema et al., 2013). As one experiences further benefits through the newly acquired PEB, such as reduced stress or more free time, these positive individual experiences can strengthen the further process of internalization. Over time, as one holds on to the behavior, it might become less dependent on external factors, such as financial incentives, and more guided by growing internal reasons, that feel personally meaningful and rewarding (i.e., values around environmental responsibility), which can in turn function as future determinants of potential engagement in PEB (Masson and Otto, 2021). This illustrates the gradual shift from externally to a more internally regulated motivation (i.e., internalization). However, the different stages of extrinsic motivation are not necessarily opposing forces. Rather, external incentives can serve as an entry point for more internalized forms of motivation, as individuals come to value the underlying environmental goals (Bardach and Murayama, 2025). Thus, the challenge lies less in avoiding external regulated motivation altogether than in designing strategies that facilitate its internalization over time. In other words, when PEB is no longer perceived as a burdensome obligation or a loss of comfort, but as an expression of one's beliefs and values, through further internalization, the likelihood of long-term behavioral change and implementation of new habits increases (Legault, 2023; Cooke et al., 2016). Hence, living out one's own internal beliefs and values offers the opportunity to turn the behavior into something experiential and beneficial right here and right now, meeting the psychological barriers in the context of environmentalism (Dioba et al., 2024; Cooke et al., 2016).

This journey from external to more internalized motivation is at the heart of Ryan & Deci's *Organismic Integration Theory* (OIT), a sub-theory of *Self-Determination Theory* (SDT) (Deci and Ryan, 1985). OIT describes extrinsic motivation as a continuum consisting of four types of regulation, which vary according to the degree to which motivation originates from external or internal factors. The process of shifting from highly external to predominantly internal motivational factors is known as internalization, whereby externally motivated behaviors can gradually become more autonomous, self-determined, or even (but not necessarily) intrinsically motivated. Regarding PEB, recent studies show that individuals with stronger internal motivation practice more challenging (Aitken et al., 2016; Green-Demers et al., 1997), enduring and frequent (Lavergne et al., 2010) PEB compared to individuals with low and rather external motivation (De Groot and Steg, 2010; Legault, 2023; Pelletier et al., 2011; Green-Demers et al., 1997; Lavergne et al., 2010). This relationship has been demonstrated within various domains, as well as within various settings. Moreover, high internal motivation appears to reduce inconsistencies associated with PEB, as individuals respond more strongly to potential inconsistencies by making more effort to adapt their motivation to their behavior (Lavergne and Pelletier, 2016). However, research specifically addressing whether increased internalized motivation leads not only to more frequent PEB but also to the spread of PEB into additional, more diverse sectors of everyday life, such as nutrition, mobility, or consumer practices, is still limited. Further insights, demonstrating that internalization fosters a spread of PEB into more diverse sectors of everyday life, could provide a rich understanding of the motivational processes that underpin long-term commitment to environmentalism. This could, for example, advocate for the design of measures that support individuals on their journey toward internalization, ultimately paving the way for PEB to be experienced not as a restrictive loss of comfort but as something personally meaningful in the long run (Maki et al., 2019), representing a worthy reward.

Hence, based on OIT, the present study follows the question whether higher internalized motivation, predominantly driven by internal factors such as personal values and beliefs, is not only associated with an increased frequency of PEB but also with a broader spread of such behavior across different sectors of everyday life. In the further course of the study, “sectors” refer to specific areas within the private household domain in which behavior can be enacted, such as mobility, energy saving, waste management and recycling, consumption, nutrition, or social engagement. These behaviors are considered within the broader context of environmentalism.

In the following, we first outline the theoretical background of OIT, discussing its advantages over established theories such as the *Theory of Planned Behavior* (Ajzen, 1985), the added value of the process of internalization for assessing the quality of PEB, and current research on the role of pro-environmental motivation. We then describe the methodology of the present study, including the rationale for recruiting participants from the Global North (Lees, 2021), the calculation of the main variables *Spread of PEB* and *Regulation Type*, and the structure of the four-part questionnaire. Specifically, participants completed a questionnaire assessing their degree of internalized motivation for PEB in everyday life, followed by indicating whether and how frequently they engaged in PEB across six specific sectors of everyday life. Next, we present and discuss the statistical results and exploratory analyses. Finally, we elaborate on theoretical and practical implications of our study while also reflecting on conceptual and methodological limitations.

## 2 The motivation for pro-environmental behavior

In the following section, we highlight the importance of examining internal factors related to environmentalism, particularly the underlying motivation for PEB. We discuss how a highly internalized pro-environmental motivation (PEM), such as a strong pro-environmental self-identity, can improve the prediction of PEB, but does not fully explain how PEB can be driven by both external and internal motivational factors, nor the qualitative differences that may exist between the two. The section concludes with an explanation of OIT, which conceptualizes extrinsic motivation as a continuum ranging from external to internal regulation, thereby offering a framework to account for qualitative differences between less and more internally driven forms of motivation.

### 2.1 The role of internal factors in explaining pro-environmental behavior

Explaining PEB has been a central focus in behavioral research, with numerous theories examining its key determinants. One influential example is Ajzen's *Theory of Planned Behavior* (TPB) (Ajzen, 1985), which is often used as a foundation in environmental psychology. TPB seeks to predict behaviors that result from planned considerations and evaluations of their consequences (Graf, 2007). It views individuals as rational beings who make decisions by systematically weighing available information. TPB identifies three core predictors of intention: *attitude* (evaluation of the behavior's outcomes), *subjective norms* (perceived social pressure), and *perceived behavioral control* (belief in one's ability to perform the behavior under given constraints). The interplay of these factors, evaluated by an individual that is confronted with the information in a specific situation, affects the *intention* to ultimately perform a behavior (or not) (Ajzen, 1991; Lange, 2010). While TPB offers a structured understanding of intention formation, it does not account for the deeper, internal processes that influence how individuals interpret and weigh information in decision-making, such as PEM.

Therefore, for instance, Whitmarsh and O'Neill (2010) included pro-environmental self-identity, the extent someone sees themselves as a type of person who acts in an environmentally friendly way as an internal factor, showing that individuals with a stronger self-identity were more likely to engage consistently in PEB. Although Whitmarsh and O'Neill's findings highlight the importance of considering internal factors for understanding individuals' PEM, self-identity alone does not capture the full range of motivational influences. It reflects only one, highly internalized level at which behavior is primarily shaped by internal factors. However, PEB can also be determent by external factors such as tax reductions or financial incentives, reflecting non-environmental reasons (Kollmuss and Agyeman, 2002; Legault, 2023). Therefore, the motivation for PEB cannot be fully explained and covered by the strength of one's self-identity alone, but rather by the degree to which motivation is regulated by rather internal vs. external factors.

### 2.2 Extrinsic motivation as a continuum of internalization

**Figure 1:**
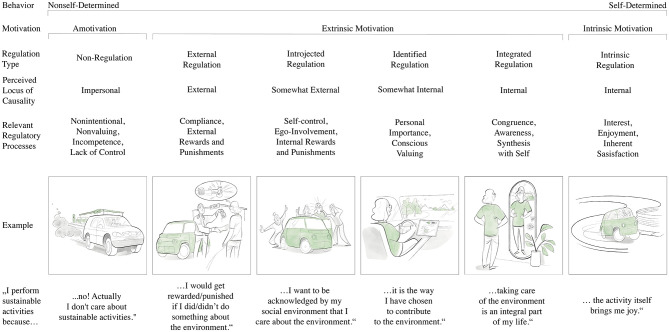
The Self-Determination Continuum Showing Types of Motivation with Their Type of Regulation, Perceived Locus of Causality, and Corresponding Process of Organismic Integration Theory based on Ryan & Deci (Ryan and Deci, 2000), extended with an Example of the Development of the Pro-Environmental Behavior Driving an Electric Car. Illustration: Frank Josten/frankjostenstudio.com.

Here, OIT (Deci and Ryan, 1985) provides a more nuanced framework by conceptualizing motivation along a continuum of internalization, capturing the varying degrees to which extrinsic motivation is gradually adopted until PEB occurs from internalized regulation and coincide with one's internal personally endorsed values and life goals (De Groot and Steg, 2010; Hurst et al., 2013). The more internalized the motivation, the more desired and sustained the behavior exhibited and the more autonomous people feel in order to flourish eventually (Deci and Ryan, 1985; Ryan and Deci, 2000; Cooke et al., 2016; Gerstenberg et al., 2024; Reeve, 2012). This perspective enables a deeper understanding of the quality of motivation that underlies PEB. As part of SDT (Deci and Ryan, 1985), which distinguishes between three main types of motivation, (1) amotivation, characterized by a lack of motivation; (2) extrinsic motivation, engaging in a behavior for instrumental reasons, meaning the behavior is performed to attain positive or avoid negative outcomes and (3) intrinsic motivation, meaning that the behavior is performed out of pure joy in the behavior itself, OIT focuses on the core in the middle of the continuum. Extrinsically motivated behaviors vary in their personal endorsement and degree of autonomy with which it is regulated, ranging from external regulation, where behavior is controlled by external rewards or punishments, over introjected and identified to integrated regulation, where behaviors are fully internalized and aligned with personal values. This continuum of extrinsic motivation makes OIT (Deci and Ryan, 1985) a very differentiated construct, highly relevant to the context of sustainability, which, as described, is both motivated by external incentives and by personal values. The different forms of motivation of SDT and regulation types of OIT are outlined in the following according to Figure 1.

#### 2.2.1 Amotivation

Amotivation is a state of complete lack of motivation, characterized by an absence of intention, purpose, or perceived control over one's behaviors. Individuals experiencing amotivation do not see value in a behavior, feel incapable of performing it effectively, or doubt that it will lead to any meaningful outcome. As a result, their actions may be disengaged, mechanical, entirely absent, or given up, and often accompanied by feelings of alienation. Amotivation stands at the lowest end of the self-determination continuum, lacking both intrinsic and extrinsic motivation, without any feelings of self-regulation, and is closely related to learned helplessness (Abramson et al., 1978), where individuals feel powerless to influence their circumstances. For example, imagine a person sitting in their combustion engine car and driving through the city. They drive past an electric car dealership without paying any attention to it, as this has no personal significance for them, they do not actively engage with the meaning or could not see what was in it for themselves, not to mention making a connection between their behavior and the consequences for the environment.

#### 2.2.2 Extrinsic motivation

Extrinsic motivation refers to engaging in a behavior for instrumental reasons, meaning the behavior is performed to attain positive or avoid negative outcomes rather than for its inherent enjoyment. Extrinsically motivated behaviors vary in their personal endorsement and degree of autonomy with which it is regulated, ranging from external regulation, where behavior is controlled by external rewards or punishments, over introjected and identified to integrated regulation, where behaviors are fully internalized and aligned with personal values. This continuum of extrinsic motivation makes OIT to a highly differentiated construct, compared to its outer poles amotivation and intrinsic motivation.

##### 2.2.2.1 External regulation

External regulation, as the least internalized and self-determined form of extrinsic motivation (De Charms, 1968), refers to behaviors driven primarily by external demands, rewards, pressure or punishments rather than personal value and endorsement or internalized interests and goals. In this “in order to” type of motivation, individuals engage in behaviors to obtain incentives, avoid penalties, or meet external expectations, often experiencing their actions as controlled or alienated. Rooted in operant conditioning (e.g., Skinner, 1953). For example, if you imagine again the person who now decided to buy an electric car from the car dealer because they receive a government subsidy or tax break or will avoid a penalty such as a CO_2_ tax.

##### 2.2.2.2 Introjected regulation

Introjected regulation is another form of extrinsic motivation in which individuals begin to internalize external pressures but do not fully accept them as their own. This type of motivation is characterized by behaviors driven by self-imposed contingencies, such as avoiding guilt or anxiety and seeking self-worth or pride. While introjected regulation represents a shift toward internal control and an increase in self-determination compared to external regulation, it remains a controlled form of motivation, as determining factors remain somewhat external to the self. Thus, external and introjected regulation have been combined to a form of controlled motivation in some studies (Williams et al., 1996). Individuals act out of a sense of obligation, striving to maintain regulation by self-esteem or gain social approval, rather than out of genuine personal endorsement. By exposure to social evaluation processes rewards for the actual behavior become immaterial and experiential, rather than material and countable. For example, you could again observe the person arriving in a social situation in the new electric car. Everyone congratulates them for driving such an environmentally friendly car. Something they expected to happen when they bought the electric car and something they are very proud of.

##### 2.2.2.3 Identified regulation

Identified regulation is the next form of extrinsic motivation in which individuals willingly engage in a behavior because they recognize its personal importance and relevance to their goals. Unlike introjected regulation, where behaviors are driven by internal pressures such as guilt or self-esteem maintenance, identified regulation involves a conscious valuing of the behavior, leading to a greater sense of choice and autonomy, and leading to a somewhat internal perceived locus of causality. Although the behavior remains instrumental, external motives have been sufficiently internalized, making the behavior feel personally meaningful as the individual consciously chooses to engage in it. This increased alignment between external demands and individual goals enhances self-determination, allowing individuals to experience a greater sense of ownership and commitment in their actions. Think again about the person sitting in the electric car while driving through the countryside. The display shows the low energy consumption of the own journey. The person appreciates the clean surrounding, which is very valuable to them now and in the future and acts as a motivator for showing the PEB.

##### 2.2.2.4 Integrated regulation

Integrated regulation is the most internalized form of extrinsic motivation, occurring when an individual fully assimilates an externally motivated behavior into their self-concept. In this process, identified regulations are evaluated, aligned, and brought into coherence with other personal values, needs, and goals, creating a deep sense of volition, personal endorsement and psychological freedom. Although the behavior remains instrumental and extrinsic, performed to achieve separable outcomes rather than for the sake of it, it is experienced as an authentic expression of the self. This high level of internalization reduces any sense of external control or obligation, making the behavior feel self-directed, self-chosen and personally meaningful, closely approximating intrinsic motivation in terms of self-determination and commitment. If we look again at the person and their electric car, we can recognize a big change. The person is standing in front of a mirror, looking at themselves. There is a sticker on the mirror with the words “Planet Earth”. We also see the electric car reflected in the person's back, which is parked in the driveway. All these details reveal that the person has a high pro-environmental self-image, because being environmentally conscious in their behavior has become a fundamental part of who the person is.

#### 2.2.3 Intrinsic motivation

Intrinsic motivation is the most self-determined form of motivation, characterized by engaging in a behavior purely for the inherent pleasure, satisfaction, and interest it provides. Unlike extrinsic motivation, where behaviors are driven by positive or negative outcomes, intrinsically motivated behaviors are performed as a reward and for joy in themselves, without any instrumental purpose (even if these can theoretically be represented, they do not form the prerequisite). Especially in the context of PEB, this condition poses a particular challenge for intrinsic motivation, although it may not necessarily be of great relevance here (Steg et al., 2016; Van Der Werff et al., 2013), precisely because pursuing moral values or personal goals take a back seat. This form of motivation fosters a deep sense of autonomy and competence, performed out of personal choice. Because intrinsic motivation arises naturally from curiosity and enjoyment, external rewards or threats can sometimes even undermine it by shifting the focus away from the activity's inherent value (Deci and Ryan, 1985). Returning to the person in the electric car, imagine how the person enjoys the driving experience of the electric car – the quiet motor, the instant torque, the innovative technology – and feels an inner sense of pleasure from just driving the car. The behavior is rewarding in itself and reflects their personal interest.

This progression from amotivation to intrinsic motivation, as outlined in OIT, illustrates how motivation is not a fixed state but a dynamic process of internalization. Especially within the degrees of extrinsic motivation, OIT provides a nuanced understanding of how behaviors can become increasingly internalized and self-determined as external regulations are gradually adopted and integrated into one's sense of self. This continuum, from externally controlled to fully internalized forms of motivation, captures the varying degrees of autonomy, each associated with qualitatively different experiences of regulation. By focusing on these differences, OIT offers a valuable framework for understanding the diverse drivers of PEB and how they evolve across the spectrum of internalization.

### 2.3 The qualities of highly internalized motivation

Beyond the different experiences, the progression of internalization is accompanied by another differentiation, regarding its quality. Highly internalized forms of motivation such as identified and integrated regulation are associated with greater behavioral persistence and higher frequency compared to rather external forms of motivation such as external and introjected regulation as individuals engage in behaviors not out of obligation, but because they are personally meaningful and aligned with self-endorsed values (De Groot and Steg, 2010; Legault, 2023; Pelletier et al., 2011; Green-Demers et al., 1997; Lavergne et al., 2010; Webb et al., 2013).

Whitmarsh and O'Neill (Whitmarsh and O'Neill, 2010) suggest that people who see themselves as environmentally conscious make consistent pro-environmental choices even across sectors. However, Ryan and Deci (Deci and Ryan, 1985) underline that this is highly dependent on the type of regulation. For example, if an individual engages in a certain PEB in sector A because it is socially accepted, the person is more likely to act out of an introjected regulation, that is an adoption of social norms without full identification with the PEB itself. In this case, according to the authors (Deci and Ryan, 1985), it is unlikely that the individual will also make pro-environmental decisions in sector B. Though, if the PEB is based on a rather higher level of internalization, i.e., is experienced as personally meaningful and autonomous it might be the case, that PEBs will also be transferred to other areas of everyday life.

In sum, OIT provides a more nuanced framework for understanding the dynamic transformation of regulation and its impact on the prediction of PEB across sectors. However, even if the fundamental relationship between internalized motivation and PEB has been demonstrated across various sectors (Legault et al., 2022; Webb et al., 2013; Schösler et al., 2014), including energy conservation, pro-environmental purchasing, or food choices, as well as across various domains (Reeve, 2012; Grolnick and Ryan, 1989; Kiliç et al., 2025; Saini et al., 2025) such as the workplace, home, and educational institutions, findings regarding the effects on the spread of PEB across several sectors in particular are rather poor. Studies mainly focus on the frequency of the performed behavior, showing that higher internalized motivation leads to more frequent PEB, which indeed occurs in different sectors of everyday life, but the consideration of a general spread across the different sectors caused by a high internalized motivation is rarely examined in particular. For example, Osbaldiston and Sheldon (Osbaldiston and Sheldon, 2003) investigated the processes by which people internalize new PEBs. Although these behaviors could originate from different sectors, there was no systematic analysis or specific evaluation of the represented environmental sectors in which the participants were active, similar to comparable studies. The authors also stated that their “study does not provide direct evidence on the issue of whether the behaviors were generalized to other domains” (Osbaldiston and Sheldon, 2003). However, this might be of great importance, because it reflects the depth and transferability of internalized motivation, especially important for environmentalism. Here, many policies and campaigns target single behaviors (e.g., recycling, using public transport). If internalized motivation can spread (Nash et al., 2017; Steinhorst et al., 2015; Truelove et al., 2014) across divers sectors (e.g., sustainable food choices, energy conservation), it would increase the efficiency and long-term impact of interventions.

Research into environmentalism, with PEB spreading across various sectors has yielded significant results in relation to a variety of influencing factors (Maki et al., 2019), including feelings of guilt, the degree of difficulty, and the presence of similarities in the behaviors concerned. However, this research has predominantly been conducted within a single area of life (Gao et al., 2017; Perrin and Barton, 2001; Sintov et al., 2001; Xu et al., 2021), thereby still leaving lacks in our understanding of the mechanisms through which PEB spreads across different areas of life, such as behavior in the private household spreading into behavior at work or in sports clubs (Nash et al., 2017; Mi et al., 2024).

A number of studies have examined the impact of motivation, distinguishing between internal and external factors, such as environmental vs. monetary framing (Steinhorst et al., 2015). These studies have demonstrated that interventions targeting intrinsic motivational elements can spread across divers sectors regarding behavioral intention (Nash et al., 2017; Truelove et al., 2014). However, these interventions are frequently confined to the one PEB, such as recycling, on another PEB, such as energy, and without considering the cross-life domain aspect (Maki et al., 2019). Most importantly, none of them do incorporate self-determined motivation according to the perspectives of OIT, which further confirms that this aspect should receive greater attention in future research.

Overall, considering internal factors appears essential for understanding PEM, but motivation exists along a continuum between external and internal regulation. While OIT explains how internalized motivation can lead to more frequent and consistent PEB, its potential to examine spreading across several sectors in particular remains underexplored. Understanding this spread could reveal how deeply rooted internalized motivation supports broader environmental engagement.

## 3 Materials and methods

The current study follows the question to what extent internalized motivation, predominantly driven by internal factors such as personal values and beliefs, is not only associated with the frequency of PEB but also with the spread of such behavior across different sectors of everyday life. It thereby aims at advancing the understanding of PEB addressing the mentioned lack of research through the lens of OIT (Deci and Ryan, 1985; Ryan and Deci, 2000) and focuses on validating the relationship between pro-environmental attitudes, the presence of PEB in everyday life and primary across divers sectors, and the level of PEM as described by OIT (Deci and Ryan, 1985; Ryan and Deci, 2000). By looking at how these factors correlate with each other, the study aims to hint at new understandings regarding the PEB of private households spreading across divers sectors of everyday life that are worth exploring further in terms of higher levels of self-determined PEM. This foundation is critical, as it underscores the importance of understanding the mechanisms driving motivational transformation. To address our research question, we conducted an online survey, where relevant variables were assessed.

### 3.1 Aim and hypotheses

Based on the above-elaborated theoretical framework and previous empirical findings, we derived the following hypotheses^1^:

1) The higher the participant's levels of motivational regulation the more positive their pro-environmental attitude. This hypothesis is grounded in previous assumptions that attitudes reflecting pro-environmental attitudes are closely aligned with more autonomous forms of motivation (Deci and Ryan, 1985; Ryan and Deci, 2000), such as identified or integrated regulation (Legault, 2023; Osbaldiston and Sheldon, 2003; Kaiser et al., 2010; Pelletier et al., 1998).
2) The higher the participant's levels of motivational regulation the more frequent they engage in PEB in their everyday lives. This hypothesis posits that the enactment of PEB reflects deeper internalization (Deci and Ryan, 1985) of pro-environmental values, aligning with higher regulation levels (De Groot and Steg, 2010; Legault, 2023; Cooke et al., 2016; Van Der Werff et al., 2013).
3) The higher the participant's levels of motivational regulation the broader they engage in PEBs that spread across their everyday life sectors. This hypothesis suggests that spread in PEB indicates a more comprehensive integration of environmentalism into the individual's identity (Whitmarsh and O'Neill, 2010; Thøgersen and Ölander, 2003), corresponding to more autonomous forms of regulation.

### 3.2 Participants

Participants in the present study, recruited through the online recruitment platform Prolific (Prolific, 2024), were paid 2.94 Euros for completing the 17-min survey in order to meet the national minimum wage of 12.41 Euros per hour (in 2024). As countries in the Global North are responsible for the highest CO_2_ emissions per capita worldwide (Ritchie et al., 2023), they were chosen as the target group for the sample. Therefore, people were included who live in a country that belongs to the Global North according to the so-called *Brandt Line*. “The Brandt Line is a way of visualizing the world that highlights the disparities and inequalities between the wealthy North and the poorer Global South.” (Lees, 2021). This includes, for example the United States (16.5 t CO_2_/pc), Canada (12.9 t CO_2_/pc), Europe (7.8 t CO_2_/pc), Australia (12.9 t CO_2_/pc) and New Zealand (7.6 t CO_2_/pc) (Ritchie et al., 2023). In addition, it was required that the participants were fluent in English.

We used the software program G^*^Power to conduct an a priori power analysis. To ensure sufficient sensitivity, we assumed a small-to-medium expected effect size of 0.20 and aimed to obtain 0.80 power at the standard α = 0.05 error probability. Based on this, a total sample size of 153 participants was required. Although the final sample consisted of only *N* = 146 participants, the observed correlation between *Regulation Type* and *Spread of PEB* in the main analysis was higher than expected (*r* = 0.34), resulting in a substantially higher achieved *post-hoc* power of 0.99.

The final sample (*N* = 146) included 67 male (45,9%) and 79 female (54,1%) participants, ranging between 19 and 61 years (*M* = 32.8, *SD* = 9.7). The stated place of residence can be divided into Europe (90.3%), Canada (4.1%), United States (2.8%), Australia (2.1%) and Japan (0.7%). The reported professional status was (self-)employed full- or part-time (71,2%), student (19,2%), unemployed (3,4%), job seeking (2,1%), unable to work (2,1%), housewife^*^husband (1,4%) or retired (0,7%). The participants stated that their highest vocational qualification was a bachelor's (37,7%) master's (36,3%), or high school degree (21,2%), 3,4% have a doctorate and 1,4% less than a high school diploma. 50% reported an income of €1,500 to €4,000, while 18.5% were below this and 31.5% were above. In addition, information was requested regarding the participants' living, relationship and family status, which are presented in the Appendix Table 1.

### 3.3 Measures and procedure

All measures utilized in the study, were collected via an online survey hosted on the platform SurveyMonkey (SurveyMonkey, 2024), to assess key constructs and the variables of interest. The selection of these measures was informed by their relevance to the research objectives, their reliability and validity in prior studies, and their suitability for an online research context. The study was pre-registered in the Open Science Framework (registration DOI: https://doi.org/10.17605/OSF.IO/VWD6J) before data collection began. Furthermore, an ethics application was submitted to the data protection officer at the University of Siegen, and the safety of conducting the study was obtained. Lastly, all participants were provided with comprehensive information regarding the study prior to its commencement and informed consent was obtained.

#### 3.3.1 Motivation toward the environment scale and relative autonomy index

To identify the *Regulation Type* of participants in the context of their PEB, we employed The *Motivation Toward the Environment Scale* (MTES) (Pelletier et al., 1998), measuring the motivational constructs proposed by Deci and Ryan (Deci and Ryan, 1985) on six subscales, and calculated the results using the *Relative Autonomy Index* (RAI) (Grolnick and Ryan, 1989; Connell and Ryan, 1986) weighting. This measure enabled us to determine participants' motivational orientations, ranging from extrinsic to intrinsic, regarding PEB. Participants were prompted with the following initial question: “Why do you or do you not perform sustainable activities in your everyday life?”. Responses were recorded on a 5-point Likert scale (ranging from 1 = does not apply at all to 5 = does apply very much). The current study demonstrated that all of the six *MTES* subscales had high levels of internal consistency (0.78 to 0.87) and an overall internal consistency of *Cronbach's a* = 0.888. These results are in line with previous studies (Pelletier et al., 1998).

#### 3.3.2 Pro-environmental attitude and exposure

To evaluate participants' *Pro-Environmental Attitude*, we adapted several subscales from Metag and colleagues (Metag et al., 2017), who aimed to identify attitudes toward climate change among the German public. The following subscales were included: Beliefs about Climate Change, General Environmental Awareness, Urgency of Collective Action, Value Dimensions, parts of Concern about Climate Change and Knowledge of Climate Change. Participants rated their agreement with a series of statements on a 5-point Likert scale (ranging from 1 = completely disagree to 5 = completely agree). The scale showed an overall internal consistency of *Cronbach's a* = 0.894 across all selected subscales.

Two items from “Concern about Climate Change” were slightly modified and combined to create the variable *Exposure to Climate Change*. The adapted items were: “I am personally affected by the worst impacts of climate change” (ranging from 1 = not at all to 5 = extremely) and “I live in a region which has been most negatively impacted by climate change” (ranging from 1 = very far away to 5 = in the immediate vicinity). Although the overall internal consistency was modest (*Cronbach's a* = 0.684), the inter-item correlation was substantial (*r* = 0.53, *p* < 0.001), indicating that both items assess related aspects of *Exposure to Climate Change*. Importantly, the central findings of the study do not rely on this measure alone. To address potential concerns regarding reliability, sensitivity analysis were conducted by analyzing each item separately regarding the main variables instead of using the composite score. This is presented in the Appendix Table 7 and will be further addressed in the discussion.

#### 3.3.3 Frequency and spread of PEB

To measure the *Frequency of PEB*, we asked the participants to evaluate their current engagement across six sectors adapted by Kaiser (2020): mobility, energy saving, waste management and recycling, consumption, nutrition, and social engagement. For each of the sector a short definition and a few behavior examples have been giving, strengthening the participant's understanding, before asking for their frequency. For example, for the mobility sector this was as follows: “Mobility in the household sector under the aspect of sustainability refers to the adoption and promotion of transportation methods that minimize environmental impact, energy consumption and pollution while fostering healthier and more equitable lifestyles. This includes activities such as using public transport, driving an e-car, avoiding flights…”. All of the self-generated domain definitions and examples are presented in the Appendix Table 8. Following this, participants responded to the following self-created question for each sector: “Overall, to what extent do you perform sustainable activities concerning sector x in your everyday life?”. Responses were captured on a 5-point Likert scale (ranging from 1 = not at all to 5 = extremely). Participants were able to voluntarily specify up to five behaviors for each sector they perform in their everyday lives. The variable *Spread of PEB* was generated subsequently from the data collected; this procedure is described further in section 3.4.

#### 3.3.4 Sequence effects

To control for potential sequence effects, we included two versions of the three-part questionnaire in the survey. Version 1 began with the assessment of *Pro-Environmental Attitude*, followed by the evaluation of the participant's PEB in everyday life, and concluded with the survey of their *Regulation Type*. In contrast, Version 2 was structured in the reverse order, starting with *Regulation Type*, followed by the evaluation of PEB, and ending with *Pro-Environmental Attitude*. This counterbalancing design was implemented to account for any possible biases related to the order in which the sections were presented to participants.

Additionally, the sequence in which the six everyday sectors concerning PEB were surveyed was also varied between versions. In version 1, the order of the sectors was as follows: mobility, energy saving, waste management and recycling, nutrition, consumption, and social engagement. Version 2 reversed this order, starting with social engagement and ending with mobility.

Independent sample *t*-tests revealed no significant differences between version 1 and version 2 for the variables *Pro-Environmental Attitude, Exposure to Climate Change, Regulation Type and Frequency of PEB* suggesting that the sequence of the sections and sectors did not influence the participants' responses. For example, regarding *Regulation Type, t*(144) = −0.275; *p* = 0.783; 95% CI [−0.2241, 0.1693]. However, the scale representing *Spread of PEB* showed a significant difference between versions, indicating a potential order effect on this specific variable. In response to this finding, we conducted a multivariate analysis of variance (MANOVA) with questionnaire version (1 vs. 2) as the independent variable and all five variables of interest as dependent variables. The multivariate test yielded a significant effect of version [*F(5)* = 2.61; *p* = 0.027], suggesting that the combination of variables was influenced by the questionnaire sequence. However, follow-up univariate analyses confirmed that this effect was driven exclusively by *Spread of PEB* [*F(1)* = 5.138; *p* = 0.025], whereas no significant differences were observed for the remaining variables (all *p* > 0.05). When Spread of PEB was excluded from the model, the multivariate effect was no longer significant [*F(4)* = 1.76; *p* = 0.14], indicating that a sensitivity emerged for this specific variable but no broader or systematic order effect was present (see Appendix Tables 9, 10).

Importantly, both scales *Frequency* and *Spread of PEB* are derived from the same underlying questionnaire data, differing only in their aggregation. Because only *Spread of PEB* showed sensitivity to the sequence of the questionnaire, it is unlikely that the order itself had a true content-based effect. Instead, the observed difference may reflect a statistical artifact arising from the specific calculation of the scale. Moreover, the questionnaire contained no items or instructions that would theoretically prime *Spread of PEB* in one version over the other. Thus, a genuine theoretical order effect on this measure is not plausible.

### 3.4 Analysis

We used JAMOVI 2.6.17.0 (Jamovi, 2023) to validate the data of the proposed hypotheses. The value established for testing statistical significance was *p* < 0.05. Various correlation analyses were carried out to identify significant relationships between the investigated variables: *Pro-Environmental Attitude, Frequency and Spread of PEB* in everyday life and *Regulation Type* according to OIT (Ryan and Deci, 2000). To reduce the probability of false positive findings (α error inflation), a Bonferroni correction was applied to the main analysis. It was chosen because of its conservative nature, in order to report only robust and reliable correlations. Accordingly, the significance level of the correlation analysis for testing the proposed hypotheses was lowered to *p* < 0.005.

Before the data could be used to test the proposed hypotheses, several steps were undertaken to process and categorize the raw data:

The *Pro-Environmental Attitude* scale was formed using the mean values of the surveyed items 1 to 21, which were compiled from various subscales by Metag and colleagues (Metag et al., 2017). Items 7, 8, 14, 15, and 16 were inverted prior to calculating the mean. The *Exposure to Climate Change* scale was formed using the mean values of the surveyed items 22 and 23, which were adapted from the subscale Concern about Climate Change by Metag and colleagues (Metag et al., 2017).

Next, the *Frequency of PEB* scale was formed using the mean values of the six surveyed everyday life sectors. For example, someone with the values “1, 3, 4, 3, 1, 4” regarding the question to what extent they perform PEB in the six sectors of everyday life, sustained the value 2,667 for the variable *Frequency of PEB*. Followed by that, we generated the variable *Spread of PEB* by assessing participants' engagement across the same six explored everyday sectors of PEB. For each of the six sectors, *Spread* was scored as “0” if the sector was valued as “1” (“not at all”), while a value greater than “1”, indicating that the participant is performing PEB somehow in the sector, was scored as “1”. The score of all six sectors was summed up, resulting in a potential value between 0 and 7 for *Spread*. Drawing on the same participant, they sustained the value “4” for the variable *Spread of PEB*, as they indicated PEB in four of the six sectors. This approach provides an indication of the range of PEBs practiced by the participants across the six measured sectors in their everyday life.

The formation of *Regulation Type* involved a three-step process: (1) Summary mean scores for each of the six surveyed subscales of the MTES were created representing the spectrum from *Amotivation* to *intrinsic Motivation*. (2) The RAIs (Grolnick and Ryan, 1989; Connell and Ryan, 1986) were created using participants' overall scores on the MTES (Pelletier et al., 1998). Weights were assigned to the total score of each subscale according to their ordering on the continuum of self-determination: Amotivation, external, introjected, identified, integrated Regulation and intrinsic motivation, were assigned the weights of −3, −2, −1, +1, +2, and +3. (3) RAI values were assigned to the final *Regulation Type* according to the just mentioned ordering: −30 to −20, −20 to −10, −10 to 0, 0 to 10, 10 to 20, 20 to 30.

## 4 Results

### 4.1 Descriptive data

Means and standard deviations of relevant variables are presented in Table 1. On average, the sample had a mean value of *M* = 4.16 (*SD* = 0.59; 95% CI [4.07, 4.26]) for the variable *Regulation Type*, which corresponds to *identified* (61,6%) and ranged from 3 (*introjected*, 11%) to 5 (*integrated*, 27,4%). *Amotivation, external* and *intrinsic* were not represented in the sample. Moreover, to test the proposed hypotheses, Pearson correlation analyses were carried out to explore the relationships between investigated variables: *Pro-Environmental Attitude, Frequency* and *Spread of PEB* in everyday life and *Regulation Type* according to OIT (Ryan and Deci, 2000). These are also presented in Table 1 below and will be explained in the following sections.

**Table 1:** Descriptive statistics and correlations for study variables.

**Study variables**	**Descriptives**				***Pearson's r***				
	***M***	***SD***	**95% CI of the Differences**		**1**	**2**	**3**	**4**	**5**
			**Lower**	**Upper**					
1. *Regulation Type*^b^	4.16	0.599	4.07	4.26	-				
2. *Pro-Environmental Attitude*^a^	3.88	0.527	3.79	3.97	0.360^*^	-			
3. *Frequency of PEB*^a^	2.69	0.703	2.57	2.80	0.415^*^	0.392^*^	-		
4. *Spread of PEB*^b^	4.77	1.30	4.56	4.99	0.341^*^	0.387^*^	0.844^*^	-	
5. *Exposure to Climate Change*^a^	2.46	0.790	2.33	2.59	0.086	0.289^*^	0.283^*^	0.241^*^	-

For all *df* = 144, *N* = 146.

^a^Y ∈ [1,5].

^b^Y ∈ [1,2,3,4,5,6].

^*^p < 0.001.

### 4.2 Hypotheses testing

Firstly, it was tested to what extent the participant's levels of motivational regulation and their *Pro-Environmental Attitude* are correlated. The results indicated a significant positive correlation between the participants' *Regulation Type* and their *Pro-Environmental Attitude* (*M* = 3.88; *SD* = 0.52, 95% CI [3.79, 3.97]), *r* = 0.36; *p* < 0.001, supporting the first hypothesis. This means that the higher the participant's level of motivational regulation according to OIT the more positive their *Pro-Environmental Attitude*.

Secondly, it was examined if the participant's levels of motivational regulation and the *Frequency of PEB* in their everyday lives are correlated. We found a significant positive correlation between the participants' *Regulation Type* and their *Frequency of PEB* in everyday life (*M* = 2.69, *SD* = 0.703, 95% CI [2.57, 2.80]), *r* = 0.41; *p* < 0.001, supporting the second hypothesis. This means that the higher the participant's level of motivational regulation according to OIT the more frequent they engage in PEB in their everyday lives.

Lastly, we analyzed whether the participant's levels of motivational regulation and the *Spread of PEB* across their everyday life sectors are correlated. A significant positive correlation emerged between the participants' *Regulation Type* and their *Spread of PEB* enacted (*M* = 4.77, *SD* = 1.30, 95% CI [4.56, 4.99]), *r* = 0.34; *p* < 0.001, supporting the last hypothesis and the study's primary objective. This means that the higher the participant's level of motivational regulation according to OIT the broader they engage in PEB across different sectors in their everyday lives.

### 4.3 Explorative findings

In addition to the main analyses, we investigated further correlations and descriptives regarding the *Frequency and Spread of PEB* broken down into the differentiated types of regulation and sectors of everyday life.

#### 4.3.1 Differences in frequency and spread of PEB in regulation types

**Figure 2:**
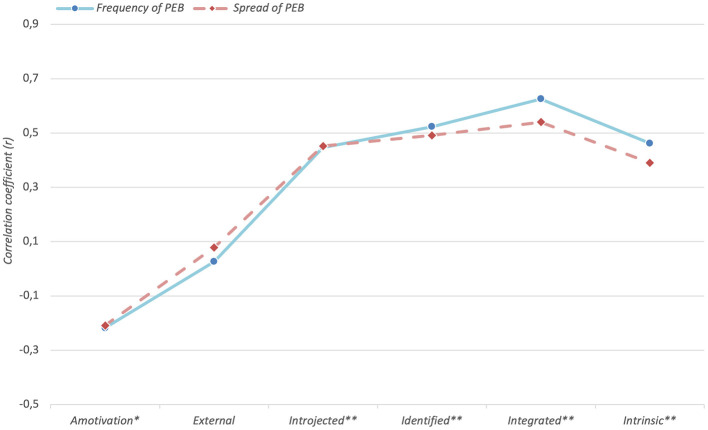
Correlations between the participants' *regulation type* and the variables *frequency and spread of PEB*. * *p* < 0.05. ** *p* < 0.001.

Descriptive results showed that 61 people (41.8%) perform PEB across all six sectors, 28 people (19.2%) across five sectors, 30 people (20.5%) across four sectors, 18 people (12.3%) across three sectors, 8 people (5.5%) in two sectors and only one person (0.7%) performed PEB in only one of six sectors. Correlations between the participants' levels of motivational regulation and the variables *Frequency and Spread of PEB* were all significant, except for the Type *external. Amotivation* correlated negatively with both investigated variables (*r*_*Frequency*_ = −0.218, *p* = 0.008; *r*_*Spread*_ = −0.209, *p* = 0.011), from i*ntrojected* to *integrated* the strength of correlation increased (*r*_*Frequency*_ = 0.44, *p* < 0.001 to *r*_*Frequency*_ = 0.62, *p* < 0.001; *r*_*Spread*_ = 0.45, *p* < 0.001 to *r*_*Spread*_ = 0.54, *p* < 0.001), before falling again in the case of *intrinsic* (*r*_*Frequency*_ = 0.46, *p* < 0.001; *r*_*Spread*_ = 0.39, *p* < 0.001) (Table 2 and Figure 2).

**Table 2:** Correlations for the *regulation types* with the variables *frequency and spread of PEB*.

**Study variables**	**Statistic**	***Regulation Type***				
		***Amotivation***	***External***	***Introjected***	***Identified***	***Integrated***	***Intrinsic***
*Frequency of PEB*	*Pearson's r*	−0.218^*^	0.026	0.447^**^	0.523^**^	0.625^**^	0.462^**^
*Spread of PEB*	*Pearson's r*	−0.209^*^	0.078	0.452^**^	0.491^**^	0.540^**^	0.390^**^

For all *df* = 144.

^*^p < 0.05.

^**^p < 0.001.

#### 4.3.2 Differences in frequency of PEB in everyday life sectors

The sample showed descriptively that the extent to which PEB is performed is strongest within the sector waste management (*M* = 3.32, *SD* = 1.04), followed by energy saving (*M* = 2.97, *SD* = 1.04), closely followed by mobility (*M* = 2.87, *SD* = 1.20) and consumption (*M* = 2.79, *SD* = 1.06), while PEB was least frequently performed in the sectors nutrition (*M* = 2.29, *SD* = 1.07) and social engagement (*M* = 1.90, *SD* = 0.952). Positive correlation between the participants' *Regulation Type* and the *Frequency of PEB* within the sectors were all significant: strongest within the sector energy saving (*r* = 0.40, *p* < 0.001), followed by waste management (*r* = 0.33, *p* < 0.001), closely followed by consumption (*r* = 0.31, *p* < 0.001), while the sectors mobility (*r* = 0.22, *p* = 0.007), social engagement (*r* = 0.21, *p* = 0.011) and nutrition (*r* = 0.19, *p* = 0.020) had the lowest correlations (Table 3).

**Table 3:** Descriptive statistics for the everyday life PEB sectors and correlations with the variable *regulation type*.

**Study variable**	**Descriptives and statistic**	**Sector**					
		**Energy saving**	**Waste management**	**Consumption**	**Mobility**	**Social engagement**	**Nutrition**
	*Mean*	2.97	3.32	2.79	2.87	1.90	2.29
	*Std.- Deviation*	1.04	1.04	1.06	1.20	0.952	1.07
*Regulation type*	*Pearson's r*	0.407^**^	0.336^**^	0.315^**^	0.221^*^	0.211^*^	0.193^*^

For all *df* = 144, *N* = 146.

^*^p < 0.05.

^**^p < 0.001.

#### 4.3.3 Exemplary qualitative profiles

Based on the values of the variables being analyzed and the participant's voluntary qualitative statements regarding the behaviors performed in their everyday life sectors, we formed exemplary low, medium and high motivation profiles of participants, representing the interplay between the investigated main variables *Frequency and Spread of PEB* and the *Regulation Types*, that are found in the sample: *Introjected, identified* and *integrated* (Table 4).

**Table 4:** Exemplary motivation profiles (low, medium, high) including qualitative statements on PEB within the six sectors of everyday life.

***Regulation type***	**Values**		**Qualitative statements**
*Introjected*	*RAI* ^a^	−6	Waste Management: “*Sorting some of my home garbage and putting it in suitable garbage bins.”*
	*Frequency of PEB*	1,16	
	*Spread of PEB*	1	
*Identified*	*RAI* ^a^	7	Consumption: “*Trying to buy more second hand and from ethical brands”*
	*Frequency of PEB*	1,83	Waste Management: “*Separating waste“, “recycling paper and plastic“, “reusing plastic bags”*
	*Spread of PEB*	4	Energy Saving: “*Turning off unnecessary electrical power consuming devices”*
			Mobility: “*Sometimes using public transportation instead of car”*
*Integrated*	*RAI* ^a^	13,5	Social Engagement: “*Voting for green policies”, “volunteering, e.g. village litter pick”, “influencing environmental policy at work”, “supporting environmental charities through social media sharing”*
	*Frequency of PEB*	3,83	Consumption: “*Buy second hand”, “prioritize experiences over consumables”, “buy local/ethical”, “choose sustainably made products”, “recycle packaging”, “repairing broken items etc.”*
	*Spread of PEB*	6	Nutrition: “*Me and oldest son are vegetarian”, “veggie meal for whole family 1-3 times/week”, “cook from scratch”, “buy local/organic when can afford it”, “milk and some yogurt delivered in glass bottles”*
			Waste Management: “*Reuse packaging, clothing etc.”, “repurpose items”, “repair items”, “compost”, “recycle”*
			Energy Saving: “*Lots of water saving - rarely flush toilet”, “green supplier of electricity and gas”, “low energy devices, e.g. washing machine”, “no tumble dryer”, “keep heating at 18 degrees in winter”, “recently increased home insulation”*
			Mobility: “*Walk or cycle where possible”, “use train over car where possible”, “drive hybrid car - good for short journeys”, “have taken 1 x flight in five years”*

^a^ Y ε [−30,30].

The participant on whose data the low motivation profile was formed, which reflects the *Regulation Type introjected* (*RAI* = −6), stated that they engage in PEB in one of the six sectors of everyday life (additionally *Frequency of PEB* = 1.16). In the sector waste management, they stated the PEB “*Sorting some of my home garbage and putting it in suitable garbage bins”*.

According to the participant whose data was used to create the medium motivation profile reflecting the *Regulation Type identified* (*RAI* = 7), they reported PEB in four of the six sectors of daily life (additionally *Frequency of PEB* = 1.83): consumption, waste management, energy saving and Mobility. Compared to the low profile, the participant indicated three PEBs for the sector waste management and said, for example, that in addition to second-hand purchases and conscious use of electrical devices, they were “*Sometimes using public transport instead of car*”.

The high motivation profile, which reflects the data of a participant who can be assigned to the *Regulation Type integrated* (*RAI* = 13.5), showed that PEB is practiced in all six sectors of daily life (*Frequency of PEB* = 3.83). Compared to the medium profile, the participant indicated multiple PEBs for all six sectors. For example, in the sector social engagement they were “*Voting for green policies”, “volunteering, e.g. village litter pick”, “influencing environmental policy at work”* and “*supporting environmental charities through social media sharing”*. In the sector mobility they stated the following PEBs: “*Walk or cycle where possible”, “use train over car where possible”, “drive hybrid car - good for short journeys”* and “*have taken 1 x flight in 5 years”*. Similarly influential measures can also be found in the other four sectors: “*prioritize experiences over consumables”, “veggie meal for whole family 1–3 times/week”, repurpose items”* and “*keep heating at 18 degrees in winter”*.

The occurrence of the individual sectors in the three *Regulation Types* was also consistent with the results of the explorative analysis regarding sector-specific correlations (Table 3). The profiles provide practical examples of the OIT continuum and show how PEB is associated with the *Regulation Types*. In doing so, they help to better understand the quantitative patterns and confirmed hypotheses.

#### 4.3.4 Correlation insights on exposure to climate change

A significant positive correlation was found between participants‘ *Exposure to Climate Change* (*M* = 2.46, *SD* = 0.79, 95% CI [2.33, 2.59]) and their *Pro-Environmental Attitude, r* = 0.28; *p* < 0.001 (Table 1). Additionally, the *Frequency of PEB* in everyday life (*r* = 0.28; *p* < 0.001) as well as the *Spread of PEB* across the everyday life sectors (*r* = 0.24; *p* = 0.003) both correlated positively with *Exposure to Climate Change*. This means that the higher the participant's level of motivational regulation according to OIT the more positive their *Pro-Environmental Attitude*, the more frequent they engaged in PEB in their everyday lives and the broader their PEB spreaded across different sectors in their everyday lives. Likewise, a significant positive correlation was found between the participants' *Pro-Environmental Attitude* and their *Frequency of PEB* (*r* = 0.39; *p* < 0.001), as well as the *Spread of PEB* across the everyday life sectors (*r* = 0.38; *p* < 0.001). It should be noted that *Exposure to Climate Change* and *Regulation Type* did not correlate significantly with each other and that the mentioned significant correlations with *Exposure to Climate Change* were descriptively rather lower than those with *Regulation Type*. This might be a sign that even if the more exposed people are to climate change it is not automatically the case that the higher their level of motivational regulation according to OIT and that the *Regulation Type* might have a stronger positive association with PEB than *Exposure to Climate Change*. We will address this further in the discussion.

## 5 Discussion

One profound approach to reinforce PEB, is to evolve from rather external regulated motivation (reward or punishment), that is always context-related and therefore not transferable to other sectors of everyday life (Legault, 2023; Stroebe, 2023), to more internal regulated motivation (as a part of identity) through the process of internalization (Legault, 2023; Deci and Ryan, 1985; Pelletier et al., 2011). Insights from the present work could provide new understandings of these motivational processes and high qualities of internal regulation that has the potential to underpin long-term commitment to environmentalism across diverse sectors of everyday life.

The hypotheses were all supported by our analyses, which sought to examine the role of OIT in relation to PEB and depicted that higher internalized motivation, predominantly driven by internal factors such as personal awareness and values, was not only associated with an increased frequency of PEB but also with a broader spread of such behavior across different sectors of everyday life.

The higher the participant's *Regulation Type*, the more positive their *Pro-Environmental Attitude* (*r* = 0.36; *p* < 0.001) and the greater the *Frequency of PEB* in their everyday life (*r* = 0.41; *p* < 0.001). By confirming these first two hypotheses, well established effects (Legault, 2023) could be replicated in a sample from the Global North, which indicates the stability of the existing relationships.

In addition, it was shown that the higher participant's *Regulation Type*, the broader their *Spread of PEB* enacted (*r* = 0.34; *p* < 0.001). This indicates that OIT most likely can be further applied to cross-sectoral behavior in the context of environmentalism. To our knowledge, the consideration of a general spread across different sectors that is related to a high internalized motivation is rarely examined in particular. Although several studies mention similar associations, unfortunately there was often no systematic analysis or specific evaluation of PEB originating from different sectors of everyday life.

### 5.1 Differences in frequency and spread of PEB in regulation types

The explorative observed pattern of correlations regarding the types of regulation according to OIT largely follows the theoretical expectation. The negative correlations between the variables *Frequency* and *Spread of PEB* and the type of regulation *Amotivation* (corresponds to the value “1”) was consistent with the idea that people reflecting this regulation type tend to report lower perceived value in PEB, feel less capable of performing it effectively, or doubt that it will lead to any meaningful outcome. Externally regulated behavior (corresponds to the value “2”) is controlled from the outside (e.g., by reward or punishment) and is therefore unstable and highly context-dependent. The result of insignificant correlations between the variables *Frequency* and *Spread of PEB* and this type of regulation are in line with the idea that external incentives alone may not be sufficient to account for consistent PEB. It is particularly noticeable that the variables *Frequency* and *Spread of PEB* were correlated the highest with the type of regulation *integrated* (corresponds to the value “5”), which indicates that the more people understanded PEB as part of their personal identity, the more frequent and the broader their PEB spreaded across different sectors. The lower correlation between the variables *Frequency* and *Spread of PEB* and the type of regulation *intrinsic* (corresponds to the value “6”) was also theoretically plausible: many PEBs are rather motivated by values, responsibility or long-term goal orientation than by immediate pleasure or interest for the behavior itself as it is the case with this type of regulation, making it more likely that PEB is shown more frequent out of personal incentives, reflecting one's self-image, than out of pure enjoyment. Furthermore, the pure enjoyment of behavior A, e.g. driving an electric car, cannot necessarily be transferred to behavior B, e.g. growing your own vegetables. Intrinsic regulated motivation is therefore, like external regulated motivation, strongly context-dependent. Thus, *Intrinsic* regulation presumably plays a lesser role in the context of environmentalism than highly internalized forms of extrinsic motivation like *integrated* regulation.

Overall, the results are consistent with the assumption of OIT that the process of internalization plays an important role in PEB and that the more pro-environmental values are integrated into the self-concept, the more consistently and comprehensively this is demonstrated. This pattern is also evident in previous research. In their *Motivational model for PEB*, Lavergne and colleagues (Lavergne et al., 2010) identified that forms of autonomous motivation (*identified, integrated and intrinsic*) have a positive effect and *Amotivation* has a negative effect on the *Frequency of PEB*, while forms of controlled motivation (*external* and *introjected*) showed descriptively positive associations but no significant effects. Webb and colleagues (Webb et al., 2013) found similar results, whereas they also worked with groupings of regulation types (controlled and autonomous) but did not analyse the six types separately. Sautkina and Ivanova (2023) analyzed the role of the different regulation types in detail, but found some deviations in the pattern along the continuum, as *introjected* regulation showed a moderately positive correlation and *identified* regulation showed weaker or non-significant correlations. The authors (Sautkina and Ivanova, 2023) justify their findings by arguing that, despite its external and therefore non-self-determined nature, *introjected* regulation can influence behavior more effectively in the short term than *identified* regulation, which is based on an awareness of the relevance of one's own behavior, through social substitutes such as guilt or a sense of duty. They emphasize that the Russian cultural context could play a role, as in a society with a strong sense of social duty, norm-based, emotionally charged motivations may have a stronger effect than individualized importance.

### 5.2 Differences in frequency of PEB in everyday life sectors

The explorative analyses showed that the degree of internalization of PEB varies from sector to sector in the present sample. Particularly in the sectors of energy saving and waste management, there was a rather strong correlation between the *Regulation Type* and the *Frequency of PEB*, which could indicate that the *Regulation Types* are more consistent, robust, predictable and less affected by variations than in the other sector specific behaviors. Both sectors are characterized by low barriers to entry, socially accepted norms and a high degree of proximity to everyday life, which simplifies the integration into personal behavioral routines and self-images. The sector of mobility showed that *Frequency of PEB* is descriptively reported similarly often as, e.g. in the sector of energy saving, but is somewhat less strongly correlated with the *Regulation Type*—which could indicate that practical reasons or infrastructural conditions might also play a role for the occurance of this behavior. Particularly in those sectors in which PEB is often characterized by higher structural, social or personal hurdles (renunciation, social norm deviation or time expenditure)— such as mobility, nutrition or social engagement—the low correlations in the present sample could indicate that PEB in these sectors is still less consistent and widespread overall and tends to occur selectively and situationally. Thus, the role of such associations needs to be explored in future research. Sautkina and Ivanova (2023) examined the role of OIT with regard to different sectors of life and found similar correlations, showing that waste management and resource conservation correlated most strongly, social behavior moderately and sustainable purchases and climate-relevant behavior (aiming to reduce carbon footprint) most weakly with high levels of regulation according to OIT. However, these results (Sautkina and Ivanova, 2023) are based on the correlations with the specific type of regulation *integrated* and a much larger sample (*N* = 462).

### 5.3 Qualitative differences between the regulation types

Based on the measured variables and the participant's qualitative statements regarding the six everyday life sectors, we formed exemplarily low, medium and high motivation profiles of participants, representing the interplay between the investigated main variables *Frequency* and *Spread of PEB* and the *Regulation Types*, that are found in the sample: *Introjected, identified* and *integrated* (Table 4).

The PEBs that were performed by the low motivation profile (*introjected*) can be viewed as low-threshold. Although it helps to conserve resources and avoid waste, its ecological effect is limited compared to other everyday sectors. The effort required for implementation and the barriers are rather low, which makes the PEB easy to integrate into everyday life. The participant may act out of a sense of obligation and strive to regulate their self-esteem or gain social approval from other potential members of their household (Deci and Ryan, 1985; Pelletier et al., 1998).

Beyond that, the PEBs in the four sectors performed by the medium motivation profile (*identified*) have different degrees of effectiveness and effort but complement each other meaningfully and effectively in everyday life. Their interplay can be plausibly assigned to the *Regulation Type identified* as, with regard to most of the behaviors, it can be assumed that they are not practiced out of external compulsion but are based on an internally accepted conviction. Even if some of the mentioned behaviors, for example in the sector mobility, are not always enjoyable and involve a loss of comfort, they might be experienced as personally valuable and relevant to their goals.

Lastly, the PEBs described by the high motivation profile (*integrated*) show a high degree of coherence, consistency and personal conviction. In their spread and intensity, they have a high ecological impact. Many of the behaviors require conscious planning, adaptation of routines or sacrifices and are accompanied by a noticeable practical and psychological effort—which speaks for a strong, internalized motivation of the participant. It can be assumed that the enduring values of these behaviors are fully integrated into the participant's self-concept. The PEBs are not only driven by external incentives, feelings of guilt or relevance to one's own goals, as is the case with the previous *Regulation Types*, but is coherent with one's own self-image and lifestyle.

The profiles provide practical examples of the OIT continuum and its qualities and show how *Frequency* and *Spread of PEB* are actually associated with the *Regulation Types*. This very stereotypical profiling based on the types of regulation represented in the sample was deliberately chosen to be as striking as possible in order to best highlight the quantitative results found regarding the variables *Frequency* and *Spread of PEB* as well as regarding the differences in the occurrence of the six sectors of everyday life.

### 5.4 Don't rely on (regional) exposure to climate change

Further correlations were exploratively investigated, focusing on the participants' *Exposure to Climate Change*. The results showed that, likewise the *Regulation Type*, participants' *Exposure to Climate Change* was positively related to *Pro-Environmental Attitude, Frequency of PEB* in everyday life as well as the *Spread of PEB* across the everyday life sectors. However, it should be emphasized that descriptively the correlations with the *Regulation Type* and the before mentioned variables were somewhat stronger than those with participants' *Exposure to Climate Change* and the mentioned variables. It was also found that there was no significant correlation between these two variables, *Exposure to Climate Change* and *Regulation Type*, which indicates that both factors might be independent from each other.

It can be said that the measure, consisting of two items, probably captures different aspects of *Exposure to Climate Change*: personal exposure (“I am personally affected by the worst impacts of climate change”) and regional exposure (“I live in a region which has been most negatively impacted by climate change”). Interestingly, personal exposure alone showed slightly stronger and more consistent associations with the investigated variables and even showed a weak correlation with the *Regulation Type*. Unlike regional exposure, which was weaker or sometimes barely not or non-significant. This pattern is consistent with the psychological distance of climate change in the Global North, where direct regional consequences are relatively rare, but personal concern may still arise through awareness of global impacts (Carmi and Kimhi, 2015; Spence et al., 2012). These findings suggest that PEB is not necessarily associated with direct regional experiences of the negative effects of climate change in your living environment. While feeling personal exposed appears to be more related to consistent and cross-sectoral PEB. Particularly regarding future challenges, it therefore seems problematic to rely on the increasing visibility and intensity of climate-related events in the Global North automatically leading to more frequent PEB among the population.

An analytical look at measured demographics, which are also influenced by more rational factors such as education (and income) have shown that there is no significant correlation with the behavioral variables and are therefore also not a reliable factor in the context of engagement in PEB. Instead, the focus should be placed more on emotional and personal valuable factors such as the motivational foundations of PEB, what seemed to be indicated by the somewhat stronger correlations between the level of regulation according to OIT and the variables *Pro-Environmental Attitude, Frequency of PEB* in everyday life as well as the *Spread of PEB* across the everyday life sectors. This form of motivation, in the sense of highly internalized and self-determined regulation, appeared to be independent of the extent to which individuals are exposed to the real consequences of climate change, indicated by the insignificant correlation between *Regulation Type* and especially the regional aspect of *Exposure to Climate Change*.

We would like to expressly point out that these conclusions are only intended as suggestions, as they were obtained based on purely correlative data. However, it could be worthwhile to empirically investigate these results in more depth in future studies, to further explore the question of whether measures to promote PEB should primarily aim to strengthen identity-related motives, rather than simply relying on the perception of the threat posed by environmental change. In addition, the different aspects of exposure should be covered by a larger item pool in order to be able to make more differentiated statements regarding their diverse associations to PEB.

### 5.5 Theoretical and practical implications

Politicians have already implemented multiple reward and punishment measures that are intended to encourage pro- and eradicate anti-environmental behavior among citizens, in the course of international agreements, such as the Paris Climate Agreement or the Green Deal within the European Union, and the achievement of climate neutrality. Whether reward or punishment measures, both can be defined as external incentives, which have some serious disadvantages compared to internal incentives in terms of PEB change in everyday life. While external incentives can indeed provide a short-term effect, they are always context-related and therefore not transferable to other sectors of everyday life (Legault, 2023; Stroebe, 2023). Thus, political measures show that their success always depends on the individual being rewarded or punished by an external incentive.

The results of the present study confirmed and extended previous research by suggesting that the focus must be on the process of internalization to better understand PEB not only in terms of frequency but also across many sectors of everyday life, bringing out the high qualities of PEB associated with OIT. Societies in which people would practice PEB out of personal values and internalized beliefs require fewer costly external incentives, thereby saving financial resources that can instead be invested in additional structural change, such as the expansion of renewable energies or public transport. One promising approach to societal change, for example, could be education for sustainable development, which strengthens environmental awareness and emphasizes the personal importance of PEB in everyday life, reflecting the core of highly internal regulation. Investing in such promotion that supports the individual journey of internalization could contribute to conditions under which lasting behavioral change in society becomes more likely. This goes beyond external incentives as the current focus of political measures and might prove to be more cost-effective in the long term.

This opens exciting areas of application, for example the domain of Human-Computer Interaction (HCI) and more particular behavior change technologies (BCTs): the aim of a BCT should be to leverage technology to support people in their process of internalization and their individual journey to develop self-regulated PEB in everyday life. In contrast to SDT as its origin, OIT has not yet been used much in BCT (Gerstenberg et al., 2024; Ryan and Deci, 2017), especially in the context of PEB (Alberts et al., 2024; Mosca et al., 2024). In the course of their review analysis, Alberts and colleagues (Alberts et al., 2024) identified 15 publications in the field of HCI that apply SDT to the design of various forms of BCTs. However, only the behavioral domains of health and education were represented, and both under the application of SDT not OIT. None of the papers identified dealt with behavior in the context of environmentalism. On the other hand, Mosca and colleagues (Mosca et al., 2024) focused in their review analysis on research that aimed to bring about a change in behavior in areas of sustainability through the inclusion of digital technologies. They identified a total of 37 studies that included the following BCTs: electricity meter system, virtual reality/immersive reality/augmented reality, gamification, persuasive mobile app, eco-driving, videos managed via the internet, computer-based learning environments, online tools and persuasive robots. The targeted PEBs were energy and water saving, reducing environmental pollution, reducing CO_2_ emissions, fund-raising and eco-literacy. None of the papers identified dealt with BCT based on SDT or OIT. OIT was first applied, still not in the context of PEB, as a basis for the design of BCTs by Gerstenberg and colleagues (Gerstenberg et al., 2024) by creating a role of an active facilitator to support people's self-regulatory transformation in terms of their health. The authors previously investigated the development of regulated motivation in connection with the process of internalization by qualitatively analyzing participant's *journey* in order to understand how people with osteoarthritis successfully introduce and maintain physical activity in the long term and out of internal regulation rather than external incentives (Gerstenberg et al., 2020).

### 5.6 Limitations and future work

Despite the informative findings regarding potential relationships that PEB span a broader range of everyday life in consideration of the regulation level according to OIT (Deci and Ryan, 1985), several limitations of the present study must be acknowledged. First, data collection relied exclusively on self-reports. While this method provides direct insight into individual attitudes and behavioral intentions, it is susceptible to biases such as social desirability and subjective misperceptions. This is particularly relevant in the context of PEB, where acting and appearing environmentally responsible is widely viewed as socially desirable (Milfont, 2009; Vesely and Klöckner, 2020). Consequently, participants may tend to present their motivation as more internalized (e.g., based on personal values) and underreport external reasons, such as acting to gain financial benefits or avoid social criticism. Such tendencies can lead to an overestimation of autonomous motivation for PEB. At the same time, a look at the descriptive data provide a more nuanced picture. Although *internal* regulation showed a relatively high mean of *M* = 3.27 (*SD* = 0.82), scores were broadly distributed across the full range (1–4.74). This indicates that *internal* regulated motivation was not necessarily uniformly high or overstated across participants. Likewise, the *introjected* regulation subscale with a mean value of *M* = 3.45 (*SD* = 0.87) suggests that motives related to social approval and internal pressure were reported openly, despite their potential social sensitivity. In addition, while mean values of amotivation (*M* = 2.10, *SD* = 0.79) and external regulation (*M* = 1.83, *SD* = 0.73) were lower, these motives were not absent. Some participants reported values above 3 and up to the upper scale range, indicating that even less socially desirable motives were acknowledged. This descriptive pattern suggests that social desirability may have influenced the responses, but there is good reason to believe that, despite the purely self-reported nature of the data, the participants' motives were not overly biased. Nevertheless, future research should address these limitations more directly by incorporating methods beyond self-reports—such as behavioral observations, experimental designs, or the inclusion of implicit measures could help reducing the influence of social desirability and provide a more accurate assessment of motivational processes.

Furthermore, qualitative elaborations regarding specific PEB were optional, which may have limited the depth and validity of behavioral assessments. From the qualitative data, it became apparent that there were a few cases where every day sectors were misunderstood, even though they were explicitly defined and examples were provided. This included mobility, which was occasionally interpreted in terms of physical activity or personal fitness, rather than in the intended context of environmentally relevant modes of transport. Those few misinterpretations may have led to the biased reporting of the *Frequency of PEB* in the sectors. However, the few cases could also be due to lack of attention, although this was controlled for with the help of two attention check items. Even though the sectors were picked based on Kaiser (Kaiser, 2020) and introduced by definitions and examples, one could question whether the six sectors can really be seen as separate as we didn't check how much the participants actually saw them as such. Future research should consider refining the sector descriptions and incorporating questions about the respondent's own understanding of the sector to confirm the psychometric separability of the sectors and to minimize interpretative ambiguity.

Second, it is important to note that the analyses are correlational in nature and do not allow for causal conclusion. Although prior research has provided evidence for positive spill-over effects to a broader range of PEB in relation to factors such as self-identity (Thøgersen and Ölander, 2003), for interventions targeting intrinsic motivations that resulted in more positive spillover for pro-environmental intentions (Maki et al., 2019), or for cross-contextual spill-over effects between PEB in households and workplaces (Mi et al., 2024), “current research does not agree on the spillover direction and driving mechanism of PEB spillover across contexts“ (Mi et al., 2024) and it finally remains unclear whether internalization according to OIT is a reliable predictor of long-term and cross-sectoral PEB. The high correlation between the variables *Spread of PEB* and *Frequency of PEB* (*r* = 0.84; *p* < 0.001) should also be noted, which could be rooted in the fact that they were interdependent as they were formed based on the same data. Future intervention studies are needed to determine the actual extent to which motivation according to OIT actually influences PEB across different sectors of everyday life and whether there is a causal effect.

Third, only three of the six regulatory types proposed by the OIT (Ryan and Deci, 2000) were represented in the sample: *introjected, identified and integrated* (corresponds to *Regulation Type* values from 2 to 4 and RAI values ranged from −7.75 to +17.5). This raises the question of whether all six forms of regulation realistically manifest in the environmental context, and whether intrinsic motivation – as defined by engaging in behavior for its inherent interest or enjoyment – is feasible or even necessary in the domain of environmentalism. In a review paper, Steg et al. (2016) examine the motivational dynamics of PEB and identify three types of goals: the hedonic goal of feeling good, the gain goal of improving one's resources and the normative goal of acting appropriately. The authors come to the conclusion that “enjoyment-based intrinsic motivation serves pro-environmental behavior best as a hedonic support of the normative goal frame from the cognitive background” (Steg et al., 2016). Another study investigated the influence of environmental self-identity and forms of intrinsic motivation on PEB (Van Der Werff et al., 2013). The authors highlighted that they did not include enjoyment-based intrinsic motivation (as opposed to obligation-based intrinsic motivation) in their analysis, as this type of motivation is of little importance in the context of environmentalism (Van Der Werff et al., 2013). While future studies should aim to include all six regulation types of OIT in their samples to obtain a complete picture of motivational dynamics regarding *Spread of PEB*, the aim could also be to identify the occurrence of the highest regulation type of OIT (*intrinsic regulation*) in the context of environmentalism.

In this study, regulation types according to the OIT were assessed using the MTES (Pelletier et al., 1998). Participants' responses were used to compute a RAI (Grolnick and Ryan, 1989; Connell and Ryan, 1986), which aggregates different regulation types into a single composite score reflecting the overall degree of motivational self-determination (ranging from −30 to +30). Based on predefined threshold values, participants were then categorized into discrete regulation types (ranging from 1 to 6), which formed the basis for subsequent analyses. While the RAI offers a valuable and theory-driven way to represent the continuum of self-determined motivation by combining multiple regulation types into a single composite score, the subsequent categorization of RAI scores into discrete regulation types introduces several methodological limitations. The transformation creates classification artifacts through unnatural threshold values, which leads to a loss of information and reduces the variance. Small differences near category boundaries (e.g., RAI scores of 9 vs. 11) may result in different classifications (*identified and integrated*), while larger differences within a category (e.g., 1 vs. 9) are ignored. This reduces conceptual accuracy and leads to a loss of statistical sensitivity. From a theoretical standpoint, the classification into ordinal regulation types aligns more closely with the OIT framework. The theory postulates a motivational continuum with qualitatively distinct regulation styles but does not assume equidistant psychological intervals between them (Ryan and Deci, 2000). On the one hand, treating the six types of regulation as ordered but not metrically scaled categories respects the conceptual structure of the theory. However, using them in Pearson correlation analyses represents a methodological inconsistency, as the analysis assumes interval scaling. This approach presents challenges because statistical analyses with ordinal variables – especially when derived from discretized data – tend to suffer from reduced variance, lower statistical power, and artifactual threshold effects. Conversely, treating the RAI as a continuous variable is methodologically advantageous. Even though it allows the use of parametric procedures such as Pearson correlations, this approach implicitly assumes that the distances between the types of regulation are psychologically equivalent – which contradicts the theoretical assumption of the theory. This trade-off was descriptively reflected in the present study, where correlations between the continuous RAI scores and the investigated variables, e.g. *Spread of PEB* (*r* = 0.41, *p* < 0.001) were somewhat stronger, than those based on the categorized Regulation Type, e.g. with *Spread of PEB* (*r* = 0.34, *p* < 0.001).

In line with this observation, Gerstenberg et al. (2020) already questioned the rigidity of the OIT's levels and emphasized that “motivation is not a particular state, but a development – a *journey*” (Gerstenberg et al., 2020). Their process-oriented perspective highlights that transitions between regulation types are fluid, overlapping, and context-dependent rather than strictly discrete. Rigid categorization by predefined thresholds may oversimplify the dynamic nature of motivational change and true complexity. It is important to understand this journey of gradual internalization, as future measures designed to facilitate it will benefit from this understanding.

So what does this individual *journey* look like in the context of PEB? How do people progress between regulation types? What are individual factors for transformation? And how can this *journey* be reinforced, for example in the form of a technology? What factors can be addressed here by applying OIT as a basis for the design of BCT to encourage PEB in its frequency and across different sectors of everyday life? Future research should consider these questions and apply additional data collection such as qualitative data to compensate for lost information and differentiations. Thereby, we could better understand the fine nuances of OIT, the relation between the qualities and more frequent and broader PEB and people's *journey* in the context of environmentalism.

## 6 Conclusion

The results of this study underlined the assumption that internalization plays a central role in relation to PEB and point to its potential relevance for behavior across different sectors. While external incentives through rewards and punishments remain a common policy tool, their limited reach and longevity stand in sharp contrast to the potential of internal incentives, like personal importance or representation of self-images. The results suggested that individuals with higher internalized and self-determined motivation, such as *identified* and *integrated* regulation according to OIT, were not only more likely to engage in PEB more frequent but did so across different sectors of everyday life. In light of these findings, it may be valuable to consider interventions, technologies and policies that not only promote short-term and context-dependent behavior change through external factors but to cultivate long-term and context-independent motivation through more internal incentives in the population and private households, as this represents a large scope of responsibility that can make a difference. Particularly in research fields such as BCT, the integration of such findings based on theoretical considerations of motivation such as OIT can promote the individual process of internalization and, in turn, could relate to meaningful reductions in household-related environmental impact in everyday life.

Jane Goodall, behavior scientist and environmental activist has hope, as “we are finally beginning to use our intellect to come up with technological solutions that will enable us to live in greater harmony with our planet […] and to think about our own ecological footprints […] Every single day that we live, we make some impact on the planet. We have a choice as to what kind of impact that is.” (Walsh, 2019).

## Data Availability

The original contributions presented in the study are included in the article/supplementary material, further inquiries can be directed to the corresponding author.

## Footnotes

1 The final wording of the hypotheses differs from that in the pre-registration, as we considered it more appropriate to focus on the regulatory level and place it at the forefront, but the content is essentially the same: 1) The more positive the sustainable attitude of the participants, the higher their regulation level. 2) The more sustainable activities the participants perform in their everyday lives, the higher their regulation level. 3) The broader the sustainable activities of the participants across their everyday live sectors, the higher their regulation level.
