# Supplementary material for: Going green from within: correlational insights into the spread of pro-environmental behavior through the lens of organismic integration theory
Source: Front Psychol. 2025 Dec 12;16:1692227. doi: 10.3389/fpsyg.2025.1692227 (PMC12742216; doi:10.3389/fpsyg.2025.1692227)
Supplement: Supplementary file 1 [file Supplementary_file_1.pdf]

Appendix

1 Demographics

Appendix Table 1 Descriptive Statistics for Demographic Variables

Variables	Descriptives				
	M	*SD*	Min	Max	Occurrence (n/%)
8. Gender [a]	-	-	-	-	Male (*n* = 67, 45,9%), Female (*n* = 79, 54,1%)
9. Age [c]	32.8	9.70	19	61	-
10. Education [b]	3.19	0.858	1	5	Ranging from 1 = Less than a Highschool Diploma (*n* = 2, 1.4 %), Highschool Degree or equivalent (*n* = 31, 21.2 %), Bachelor's Degree (*n* = 55, 37.7 %), Master's Degree (*n* = 53, 36.3 %), till 5 = Doctorate (*n* = 5, 3.4 %)
11. Employment [a]	-	-	-	-	Student (*n* = 28, 19.2 %), House Husband/Wife (*n* = 2, 1.4 %), Employed full-time (40+h) (*n* = 81, 55.5 %), Employed part-time (<40h) (*n* = 11, 7.5 %), Self-employed (*n* = 12, 8.2 %), Unemployed (*n* = 5, 3.4 %), Incapable of Work (*n* = 3, 2.1 %), Job-seeker (*n* = 3, 2.1 %), Retired (*n* = 1, 0.7 %)
12. Income [b]	2.36	1.18	1	6	Ranging from 1 = 0 € - 1.500 € (*n* = 27, 18.5 %), 1.501 € - 4.000 € (*n* = 73, 50.0 %), 4.001 € - 7.500 € (*n* = 30, 20.5 %), 7.501 € - 10.000 € (*n* = 6, 4.1 %), 10.001 € - 15.000 € (*n* = 3, 2.1 %), till 6 = 15.001 € or higher (*n* = 7, 4.8 %)
13. Marital Status [a]	-	-	-	-	Single (*n* = 63, 43.2 %), In a Relationship (*n* = 43, 29.5 %), Married (*n* = 36, 24.7 %), Divorced (*n* = 4, 2.7 %)
14. Living Situation (how) [a]	-	-	-	-	Not specified (*n* = 7, 4.8 %), Renting a Flat (*n* = 52, 35.6 %), Renting a House (*n* = 11, 7.5 %), Owning a Flat (*n* = 30, 20.5 %), Owning a House (*n* = 46, 31.5 %)
15. Living Situation (who) [a]	-	-	-	-	Not specified (*n* = 3, 2.1 %), Alone (*n* = 34, 23.3 %), Parents (*n* = 37, 25.3 %), Partner (*n* = 29, 19.9 %), Children (*n* = 2, 1.4 %),

					Partner and Children (*n* = 31, 21.2 %), Flat-sharing Community (*n* = 7, 4.8 %), Multigenerational House (*n* = 3, 2.1 %)
16. Kids [a]	-	-	-	-	Yes (*n* = 37, 25.3 %), No (*n* = 109, 74.7 %)
17. Number of Kids [c]	2.22	2.68	1	17	-
18. Country [a]	-	-	-	-	Australia (*n* = 3, 2.1 %), Belgium (*n* = 2, 1.4 %), Canada (*n* = 6, 4.1 %), Croatia (*n* = 1, 0.7 %), Czech Republic (*n* = 2, 1.4 %), Estonia (*n* = 3, 2.1 %), Finland (*n* = 1, 0.7 %), France (*n* = 12, 8.3 %), Germany (*n* = 12, 8.3 %), Greece (*n* = 12, 8.3 %), Hungary (*n* = 9, 6.2 %), Ireland (*n* = 1, 0.7 %), Israel (*n* = 5, 3.4 %), Italy (*n* = 6, 4.1 %), Latvia (*n* = 2, 1.4 %), Netherlands (*n* = 2, 1.4 %), Norway (*n* = 1, 0.7 %), Poland (*n* = 16, 11.0 %), Portugal (*n* = 25, 17.2 %), Scotland (*n* = 1, 0.7 %), Slovakia (*n* = 2, 1.4 %), Slovenia (*n* = 2, 1.4 %), Spain (*n* = 3, 2.1 %), Sweden (*n* = 1, 0.7 %), UK (*n* = 10, 6.9 %), US (*n* = 4, 2.8 %), Japan (*n* = 1, 0.7 %)
19. English First Language [a]	-	-	-	-	Yes (*n* = 28, 19.2 %), No (*n* = 118, 80.8 %)

Note. For all $N = 146$
[a] nominal. [b] ordinal. [c] continual.

Appendix Table 2 Correlations for Demographic Variables

Study Variables	*Pearson's r*								
	1	2	3	4	5	6	7	8	9
1. Regulation Type	—								
2. Exposure	0.086	—							
3. Pro-Environ-mental Attitude	0.360**	0.289**	—						

Study Variables	Pearson's *r*								
	1	2	3	4	5	6	7	8	9
4. *Frequency of PEB*	0.415**	0.283**	0.392**	—					
5. *Spread of PEB*	0.341**	0.241*	0.387**	0.844**	—				
6. Age	0.179*	0.007	-0.072	0.166*	0.076	—			
7. Education	0.086	0.118	0.118	0.035	0.139	0.088	—		
8. Income	-0.152	-0.023	-0.092	-0.071	-0.087	-0.100	-0.082	—	
9. Number of Kids	-0.088	-0.194	-0.020	-0.127	-0.060	0.325*	-0.068	-0.270	—

Note. For all df = 144, *N* = 146
* $p < .05$. ** $p < .001$.

A look at the demographic data showed that both age and gender were associated with the main variables. The older, the higher the participant's *Regulation Type* ($r = 0.17$; $p = 0.030$) and the greater the *Frequency of PEB* in their everyday life ($r = 0.16$, $p = 0.045$). This trend is plausible, considering that older individuals are more likely to take on household responsibilities, where sustainable decision-making and behavioral routines are more directly enacted, suggesting that life stage may play an important role in the formation and expression of PEB. In the present sample, participants ranged in age from 19 to 61 years (M = 32.8, SD = 9.7).

Appendix Table 3 Independent Sample t-Test for the Variable Gender

Study Variable	Male[a]		Female[b]		t	Cohen's d
	M	*SD*	*M*	*SD*		
Pro-Environmental Attitude	3.73	0.548	4.01	0.475	-3.27*	-0.543
Frequency of PEB	15.28	4.074	16.84	4.232	-2.25*	-0.373

Note. For all df = 144, *N* = 146, [a]n = 67, [b]n = 79
* $p < .05$.

Additionally, significant gender differences were observed: Women showed a significantly more positive *Pro-Environmental Attitude* ($M = 4.01$, $SD = 0.475$) than men ($M = 3.73$, $SD = 0.548$), $t(144) = -3.27$; $p = 0.001$; 95% CI [-0.444, -0.109]. In addition, women had a greater *Frequency of PEB* in

their everyday life (*M* = 2.81, *SD* = 0.705) than men (*M* = 2.55, *SD* = 0.679), *t(144)* = -2,25; *p* = .026; 95% CI [-0.486, -0.031] and women reported more *Pro-Environmental Attitude* and greater *Frequency of PEB* compared to men. These findings align with existing literature, which consistently shows that women tend to engage in environmental activism, express greater environmental concern and are more likely to adopt PEB (1–3). Possible explanations for this effect might be gender role socialization processes, greater PEB-related traits such as altruism and empathy, and stronger normative commitments among women regarding care and safety (4,5). In addition, just as with older people, it can be assumed that female individuals are still more likely to take on household responsibilities.

2 Used Measures and Variables in the Online Questionnaire

2.1 Motivation Toward the Environment Scale *(*MTES; Pelletier et al. (6))

- 5-point Likert scale: ranging from 1 = does not apply at all to 5 = does apply very much
- Introduction: “Why do you or do you not perform sustainable activities in your everyday life?”

Appendix Table 4 Items of the MTES

Item-Nr.	Item
Amot_1	I wonder why I’m doing anything about the environment, since the situation isn’t improving.
Amot_2	I feel that doing something about the environment is a waste of time.
Amot_3	I can’t see how my efforts to be environmentally friendly are helping the environment.
Amot_4	I can’t see what’s in it for me.
Ext_1	Because other people would be mad if I didn’t do anything about the environment.
Ext_2	For the recognition I get for it from others.
Ext_3	Because my friends insist that I do it.
Ext_4	To avoid being criticized.
Intro_1	Because I think I’d regret not doing something about the environment.
Intro_2	Because I’d feel guilty if I didn’t do anything about the environment.
Intro_3	Because I’d feel bad if I didn’t do anything about the environment.
Intro_4	I’d be ashamed not to do anything about the environment.
Iden_1	Because it is a sensible thing to do something about the environment.
Iden_2	Because it is the way I have chosen to contribute to the environment.
Iden_3	Because it is a reasonable thing to do something about the environment.
Iden_4	Because I think it is a good idea to do something about the environment.
Inte_1	Because taking care of the environment is an integral part of my life.
Inte_2	Because it seems to me that taking care of myself and taking care of the environment are inseparable.
Inte_3	Because it is part of the way I have chosen to live my life.
Inte_4	Because my environmental awareness has become a fundamental part of who I am.
Intrin_1	For the pleasure I get in mastering new ways to help.
Intrin_2	For the pleasure I get in improving the quality of the environment.
Intrin_3	Because I like the feeling I get when doing things for the environment.
Intrin_4	For the pleasure of contributing to the environment.

2.2 Pro-Environmental Attitude (adapted from Metag et al. (7))

- 5-point Likert scale: ranging from 1 = completely disagree to 5 = completely agree
- Introduction: "Please rate below how strongly you agree or disagree with the following statements. There is no right or wrong."

Appendix Table 5 Items of the *Pro-Environmental Attitude*

Item-Nr.	Item
Atti_1	Climate change is man-made.
Atti_2	Climate research is of the unanimous opinion that global warming is real.
Atti_3	Climate change is currently happening.
Atti_4	Industrial countries are predominantly responsible for climate change.
Atti_5	Climate scientists can be trusted.
Atti_6	Earth's living space and resources are highly limited.
Atti_7*	The scope of the ecological crisis is being exaggerated.
Atti_8*	Earth's natural balance can withstand pollution.
Atti_9	We are heading toward an environmental disaster.
Atti_10	Human beings are damaging the environment seriously.
Atti_11	It's important to take measures against climate change as soon as possible.
Atti_12	If one acts immediately climate change can be averted.
Atti_13	We live at the expense of future generations.
Atti_14*	The enforcement of equal rights for everyone is being taken too seriously.
Atti_15*	Individual freedom is too restricted in today's society.
Atti_16*	Many problems can best be solved by individual people.
Atti_17	We are not doing enough to fight poverty in the world.
Atti_18	Climate change is a serious problem.
Atti_19	Climate change causes an increase in extreme weather events.
Atti_20	I'm seriously worried about climate change.
Atti_21[a]	How well informed are you about climate change?

Note.
*inverted Items
[a]ranging from 1 = not at all to 5 = very well

2.3 Exposure to Climate Change (adapted from Metag et al. (7))

- Expo_1
  - 5-point Likert scale: ranging from 1 = not at all to 5 = extremely
  - Introduction: "Please evaluate how strong you are affected by the worst impacts of climate change."
- Expo_2
  - 5-point Likert scale: ranging from 1 = very far away to 5 = in the immediate vicinity
  - Introduction: "Please indicate how close you live to a region that has been most negatively impacted by climate change."

Appendix Table 6 Items of the *Exposure to Climate Change*

Item-Nr	Item

Expo_1	I am personally affected by the worst impacts of climate change
Expo_2	I live in a region which has been most negatively impacted by climate change.

Appendix Table 7 Correlations for both *Exposure to Climate Change* Items

Study Variable	*Pearson's r*		
	Exposure to Climate Change	Expo_1	Expo_2
Pro-Environmental Attitude	0.289**	0.282**	0.229*
Regulation Type	0.086	0.166*	0.000
Frequency of PEB	0.283**	0.390**	0.130
Spread of PEB	0.241*	0.282**	0.152

Note. For all df = 144, *N* = 146
* *p* < .05. ** *p* < .001.

2.4 Sectors of Everyday Life (self-created, according to Kaiser (8))

- 5-point Likert scale: ranging from 1 = not at all to 5 = extremely
- Introduction: "Overall, to what extent do you perform sustainable activities concerning sector … in your everyday life?"
- Introduction for the open answers afterwards: "As you rated "slightly" or higher: Please name the sustainable activities concerning the mentioned sector, that you perform yourself (!) in your everyday life. Describing them in one short sentence is enough."

Appendix Table 8 Descriptions of the Everyday Life Sectors

Sector	Description
…Mobility	Mobility in the household sector under the aspect of sustainability refers to the adoption and promotion of transportation methods that minimize environmental impact, energy consumption and pollution while fostering healthier and more equitable lifestyles. This includes activities such as using public transport, owning an e-car, avoiding flights...
…Energy Saving	Energy saving in the household sector under the aspect of sustainability refers to the responsible use of natural resources, like water or coal, to meet household needs while minimizing environmental impact by adopting activities that optimize the use of energy. This includes activities such as saving electricity and water, taking short showers, using eco-programs, owning photovoltaic systems, not overheating rooms…
…Waste Management and Recycling	Waste management and recycling in the household sector under the aspect of sustainability involves minimizing waste generation and pollution, properly sorting materials, conserve resources and promote sustainable consumption, and reusing or recycling to reduce environmental impact. This includes activities such as separating waste, recycling plastic, glass, bottles or cans, do up-cycling, avoiding plastic…
…Nutrition	Nutrition in the household sector under the aspect of sustainability focuses on adopting eating habits and food choices that are environmentally friendly,

	socially responsible, reduce the environmental footprint and support equitable food systems. This includes activities such as reducing meat, eating veggie or vegan, considering fair trade products, buying seasonal, regional and organic…
…Consumption	Consumption in the household sector under the aspect of sustainability involves making purchasing decisions that prioritize environmental, social, and economic responsibility. This includes activities such as choosing durable, eco-friendly products, reducing consumption, avoiding overpackaging, supporting ethical brands, embracing second hand...
…Social Engagement	Social engagement in the household sector under the aspect of sustainability refers to actively contributing to the well-being of communities and fostering social responsibility by creating positive environmental and societal impacts. This includes activities such as voting for green oriented parties, volunteering and engaging in environmental protection programs, donating to ecology groups, teaching or demonstrating in terms of sustainable development…

2.5 Sequence Effects

Appendix Table 9 Independent Sample t-Test for the two Questionnaire Versions

Study Variable	Version 1		Version 2		t
	M	*SD*	*M*	*SD*	
Pro-Environmental Attitude	3.94	0.507	3.81	0.541	1.495
Exposure to Climate Change	2.50	0.750	2.42	0.832	0.575
Regulation Type	4.15	0.569	4.18	0.631	-0.275
Frequency of PEB	2.61	0.709	2.76	0.693	-1.338
Spread of PEB	4.53	1.270	5.01	1.286	-2.267*

Note. For all df = 144, for both groups *n* = 73
* $p < .05$.

Appendix Table 10 MANOVA for the two Questionnaire Versions

Modell	Statistic	Value	F
Questionnaire version[a]	Wilks' Λ	0.915	2.61*
Questionnaire version[b]	Wilks' Λ	0.952	1.76

Note.
[a]df = 5,140, including *Spread of PEB*
[b]df = 4,141, excluding *Spread of PEB*
* $p < .05$

3 References

1. Isaacson S. When do Women Take the Lead? Exploring the Intersection Between Gender Equality and Women's Environmental Political Participation from a Comparative Perspective. International Journal of Sociology. 16. Dezember 2024;54(5–6):399–423.
2. Milfont TL, Sibley CG. Empathic and social dominance orientations help explain gender differences in environmentalism: A one-year Bayesian mediation analysis. Personality and Individual

Differences. Februar 2016;90:85–8.
3. Women’s leadership in environmental action [Internet]. Paris: OECD Publishing; 2022 Apr [zitiert 6. Mai 2025]. (Smith A, Reihenherausgeber. OECD Environment Working Papers; Bd. 193). Report No.: 193. Verfügbar unter: https://www.oecd.org/en/publications/women-s-leadership-in-environmental-action_f0038d22-en.html
4. Arnocky S, Stroink ML. Gender differences in environmentalism: The mediating role of emotional empathy. Current Research in Social Psychology. Januar 2010;16(9):1–14.
5. McCright AM. The effects of gender on climate change knowledge and concern in the American public. Popul Environ. September 2010;32(1):66–87.
6. Pelletier LG, Tuson KM, Green-Demers I, Noels K, Beaton AM. Why Are You Doing Things for the Environment? The Motivation Toward the Environment Scale (MTES). J Applied Social Pyschol. März 1998;28(5):437–68.
7. Metag J, Füchslin T, Schäfer MS. Global warming’s five Germanys: A typology of Germans’ views on climate change and patterns of media use and information. Public Underst Sci. Mai 2017;26(4):434–51.
8. Kaiser FG. GEB-50. General Ecological Behavior Scale [Internet]. ZPID (Leibniz Institute for Psychology) – Open Test Archive; 2020 [zitiert 6. Mai 2025]. Verfügbar unter: https://www.psycharchives.org/handle/20.500.12034/3068.2
